# Prolonged use of Internet and gaming among treatment seekers arising out of social restrictions related to COVID‐19 pandemic

**DOI:** 10.1111/pcn.13127

**Published:** 2020-09-02

**Authors:** Susumu Higuchi, Satoko Mihara, Takashi Kitayuguchi, Haruka Miyakoshi, Madoka Ooi, Masaki Maezono, Kotaro Nishimura, Takanobu Matsuzaki

**Affiliations:** ^1^ Department of Psychiatry National Hospital Organization Kurihama Medical and Addiction Center Yokosuka Japan

The novel coronavirus disease, COVID‐19, originated in Wuhan, China, and has rapidly spread throughout the world. The World Health Organization declared the disease a pandemic on 11 March 2020. Prior to this, on 2 March, the Japanese government had requested local governments shut down elementary, and junior and senior high schools throughout the country. Other institutions, including colleges and universities, soon followed. On 7 April, the national government declared a state of emergency in selected prefectures and expanded this to all prefectures on 16 April. These measures were gradually lifted by the end of June.

During this state of emergency and the school closures, people were strongly recommended to stay at home and did so to a far higher degree than before the COVID‐19 pandemic. Social restrictions, such as stay‐home measures, would be expected to lead to an increase in the consumption of digital entertainment, particularly online gaming and related activities.^1^ Currently, empirical data showing an increase in Internet use due to social restrictions are scarce, with the exception of a small number of very recent studies.^2^, ^3^ This study explored the possible impact of these restrictions on Internet use and gaming behavior among treatment seekers with gaming disorder (GD) or excessive use of Internet/gaming (EUIG). The latter are those who use the Internet or games excessively and have related problems but have not been diagnosed as having GD.

This study was approved by the ethics committee of our center (approval No. 361), and written informed consent was obtained from all study participants, with additional consent obtained from parents where subjects were under 18 years of age. Participants numbered 80 treatment seekers with GD or EUIG who visited our center between 16 May and 12 June 2020. Almost all were male (78/80), the mean age was 18.9 years (SD, 6.4 years; age range, 12–44 years), and about 70% were school students. Seventy percent of participants were diagnosed as having ICD‐11 GD,^4^ 20% engaged in excessive gaming but were not diagnosed as having GD, and the remaining 10% engaged in excessive use of other online applications.

Upon visiting our center, psychiatrists and clinical psychologists with expertise in the treatment of behavioral addictions conducted face‐to‐face interviews using an evaluation instrument developed for this study. It contained questions pertaining to changes in Internet use and gaming, functional impairment due to GD or EUIG, and possible reasons for the change. Participants were asked about changes in Internet use and gaming behavior and the level of functional impairment between February 2020 (pre‐stay‐home period) and the 30‐day period prior to the survey (stay‐home period). Internet use for study or work activities was excluded from Internet time for the purpose of this study. The data obtained were analyzed using sas 9.4.^5^

Mean daily hours spent on the Internet, smartphones, online and offline gaming, and video viewing were significantly higher for the stay‐home period compared to the pre‐stay‐home period (Fig. 1). This was especially true for Internet and smartphone use and online gaming. Time spent on the Internet had increased between the two periods for 71.3% of participants, and 52.5% reported an increase in time spent on smartphones and online gaming. The most common reason for these increases appeared to be ‘having extended free time to use the Internet and engage in gaming due to the stay‐home measure.’ In cases where individuals had a high number of social withdrawal days in February, there tended to be limited change in time spent on the Internet between the pre‐ and stay‐home periods. In fact, repeated‐measures analysis of variance revealed that participants who were socially withdrawn for fewer than 20 days showed a significant increase in time spent on the Internet, but for those who were socially withdrawn for 20 days or more, the time spent was unchanged. ‘Social withdrawal’ is a state in which an individual stays at home, does not go to school or work, and has no direct contact with people other than the family.

**Fig. 1:**
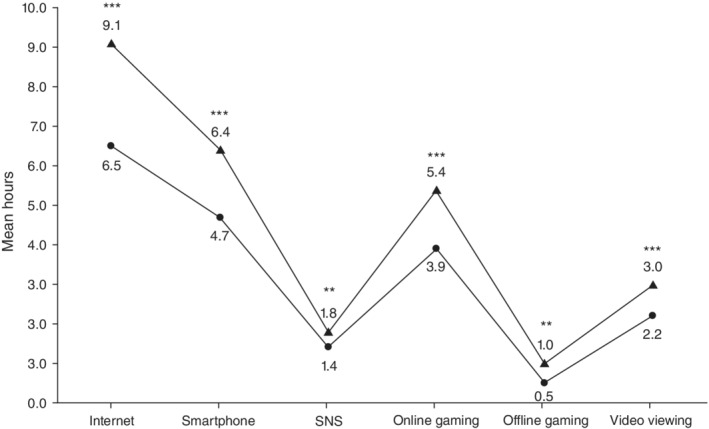
Average daily hours spent on the Internet, smartphone, social network sites (SNS), online gaming, offline gaming, and video viewing in (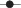) February 2020 and (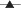) the 30‐day period prior to the survey (stay‐home period). The majority of participants used different applications simultaneously and so the figures for time spent on SNS, online gaming, and video viewing did not sum up to the time spent on the Internet. **P < 0.01, ***P < 0.001.

To better understand functional impairment due to GD or EUIG, we evaluated 10 problem areas (Table S1). Participants were asked whether each problem had worsened, was unchanged, or had improved between the pre‐stay‐home period and the stay‐home period. There was a lack of uniformity in the responses. Compared to ‘improved,’ relatively higher rates of participants selected ‘deteriorated’ for social withdrawal, sleep disturbance, difficulty waking up in the morning, day–night reversal, and insufficient physical activity. In fact, the number of days of social withdrawal was significantly higher in the stay‐home period than in the pre‐stay‐home period. Conversely, regarding the question of not studying at home, a relatively higher rate of participants responded with ‘improved’ compared to the rate for ‘deteriorated.’ This tendency was also true for participants who were socially withdrawn and did not go to school for 20 days or more in the pre‐stay‐home period, and whose situation remained unchanged at the survey conclusion. These results suggest that some participants were able to use their time at home for studying, notwithstanding the fact that most of their time was devoted to using the Internet and/or gaming. It may imply that studying at home, including time spent on online educational activities, worked better than studying at school for some of these treatment seekers.

This study suggests that social restrictions, including stay‐home measure due to the COVID‐19 pandemic, have negatively affected Internet use and gaming behavior among treatment seekers. However, this situation may also have provided an environment in which socially isolated treatment seekers were able to study more at home. This study was retrospective in design and focused only on treatment seekers. In addition, the small number of questions in the questionnaire and limited number of study participants did not allow for detailed data analysis (e.g., severity of GD/EUIG and the comorbidity of the participants). Further studies on other subject groups, including the general population and students, using a prospective design are warranted.

## Disclosure statement

All authors report no financial conflict or other relationships relevant to the subject of this article.

## Supporting information


**Table S1.** Change in the level of functional impairment due to Internet use and gaming between February 2020 and the period of 30 days prior to the survey (stay‐home period).
