# Supplementary material for: Prolonged use of Internet and gaming among treatment seekers arising out of social restrictions related to COVID‐19 pandemic
Source: Psychiatry Clin Neurosci. 2020 Sep 2;74(11):607–8. doi: 10.1111/pcn.13127 (PMC7436697; doi:10.1111/pcn.13127)
Supplement: Supplementary file 1 — Table S1. Change in the level of functional impairment due to Internet use and gaming between February 2020 and the period of 30 days prior to the survey (stay‐home period). [file PCN-74-607-s001.docx]

**Table S1.** Change in the level of functional impairment due to internet use and gaming between

in February, 2020 and the period of 30 days prior to the survey

| No | Items | Change (N=80)^a^ | | |
| --- | --- | --- | --- | --- |
|  |  | Deteriorated  (%) | Unchanged  (%) | Improved  (%) |
| 1 | Not studying at home^b^ | 8.6 | 57.1 | 34.3 |
| 2 | Social withdraw (hikikomori) | 52.5 | 37.5 | 10.0 |
| 3 | Verbal aggression to families | 15.0 | 72.5 | 12.5 |
| 4 | Physical aggression to families | 3.8 | 87.5 | 8.8 |
| 5 | Money spent on items | 13.8 | 71.3 | 15.0 |
| 6 | Sleep disturbances | 40.0 | 47.5 | 12.5 |
| 7 | Unable to wake up in the morning | 37.5 | 48.8 | 13.8 |
| 8 | Day-night reversal | 32.5 | 57.5 | 10.0 |
| 9 | Irregular meal habits | 26.3 | 62.5 | 11.3 |
| 10 | Insufficiency in physical activities | 46.3 | 38.8 | 15.0 |

^a^Change between February, 2020 and the period of 30 days prior to the survey.

^b^Missing data: Ten participants did not apply to question No. 1, because they were not students.
